# Cell and molecular targeted therapies for diabetic retinopathy

**DOI:** 10.3389/fendo.2024.1416668

**Published:** 2024-06-14

**Authors:** Shivakumar K. Reddy, Vasudha Devi, Amritha T. M. Seetharaman, S. Shailaja, Kumar M. R. Bhat, Rajashekhar Gangaraju, Dinesh Upadhya

**Affiliations:** ^1^ Centre for Molecular Neurosciences, Kasturba Medical College, Manipal, Manipal Academy of Higher Education, Manipal, India; ^2^ Department of Pharmacology, Kasturba Medical College, Manipal, Manipal Academy of Higher Education, Manipal, India; ^3^ Department of Ophthalmology, The University of Tennessee Health Science Center, Memphis, TN, United States; ^4^ Department of Ophthalmology, Kasturba Medical College, Manipal, Manipal Academy of Higher Education, Manipal, India; ^5^ Department of Anatomy, Kasturba Medical College, Manipal, Manipal Academy of Higher Education, Manipal, India; ^6^ Department of Anatomy & Neurobiology, The University of Tennessee Health Science Center, Memphis, TN, United States

**Keywords:** diabetes, anti-VEGF drugs, neovascularization, apoptosis, inflammation, blood retinal barrier, microaneurysms, leukostasis

## Abstract

Diabetic retinopathy (DR) stands as a prevalent complication in the eye resulting from diabetes mellitus, predominantly associated with high blood sugar levels and hypertension as individuals age. DR is a severe microvascular complication of both type I and type II diabetes mellitus and the leading cause of vision impairment. The critical approach to combatting and halting the advancement of DR lies in effectively managing blood glucose and blood pressure levels in diabetic patients; however, this is seldom achieved. Both human and animal studies have revealed the intricate nature of this condition involving various cell types and molecules. Aside from photocoagulation, the sole therapy targeting VEGF molecules in the retina to prevent abnormal blood vessel growth is intravitreal anti-VEGF therapy. However, a substantial portion of cases, approximately 30–40%, do not respond to this treatment. This review explores distinctive pathophysiological phenomena of DR and identifiable cell types and molecules that could be targeted to mitigate the chronic changes occurring in the retina due to diabetes mellitus. Addressing the significant research gap in this domain is imperative to broaden the treatment options available for managing DR effectively.

## Introduction

Uncontrolled blood glucose levels for extended durations are linked with multiple complications such as retinopathy, nephropathy, cardiovascular, cerebrovascular, and peripheral vascular diseases, leading to high morbidity and mortality rates with diabetes mellitus (1, 2). Diabetic retinopathy (DR) is a severe microvascular complication of both type I and type II diabetes mellitus and the leading cause of vision impairment. Recent systematic review and meta-analysis revealed that approximately 1 in 5 persons with diabetes worldwide have DR, and the total number of people losing vision as a result of DR may continue to rise (3). Multiple studies have recognized that risk factors such as severe hyperglycemia, hypertension, and hyperlipidemia worsen DR.

**Figure 1:**
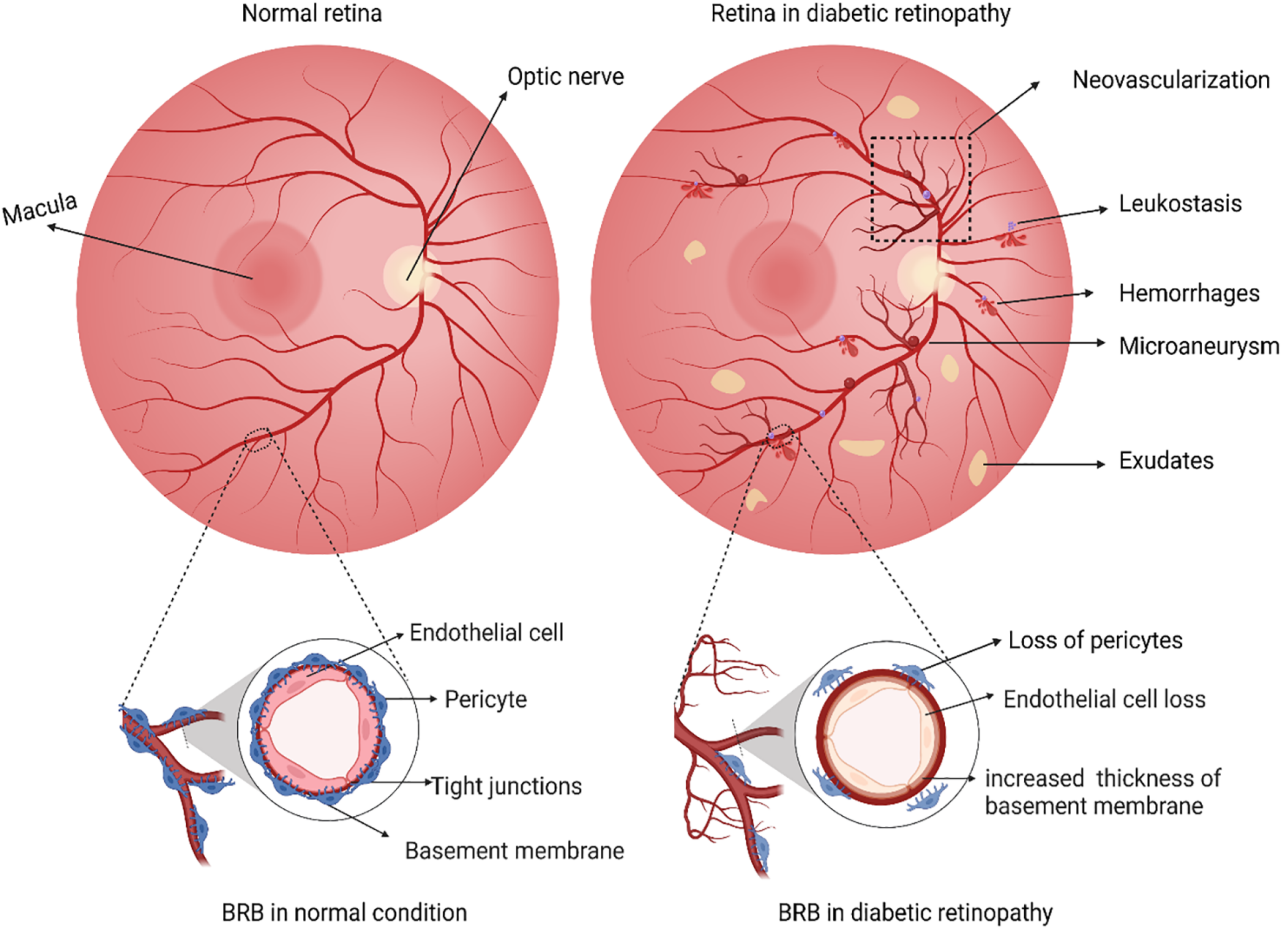
Schematic illustration representing differences between a normal retina and a retina with diabetic retinopathy. In the normal retina, a functional inner blood-brain barrier with intact endothelial cells and pericytes, while in diabetic retinopathy, dysfunction of BRB is evident through loss of pericytes and endothelial cells and thickening of the vascular basement membrane. Any loss of such retinal barrier integrity is associated with a cycle of inflammation, vascular damage, and cell death. During the retinal damage, it is likely that activated Microglia and Glial cells release high levels of pro-inflammatory cytokines. These cytokines act on nearby vasculature and neuronal cells, causing further damage. Classical pathological hallmarks of DR such as the formation of microaneurysms, abnormal growth of new blood vessels (neovascularization), microvascular leakages (cotton wool spots, hemorrhages), accumulation of thick yellowish fluids, i.e., exudates and recruitment of leukocytes due to inflammation (leukostasis) are represented. The figure was created using BioRender.

DR can be broadly categorized into background retinopathy (non-proliferative DR, NPDR) and proliferative DR (PDR) with diabetic macular edema (DME) occurring at any stage of DR. Presently, the primary treatment for NPDR involves anti-vascular endothelial growth factor (anti-VEGF) drugs targeting VEGF, a known cause of DME. However, nearly 30–40% of DR patients do not respond to these anti-VEGF treatments (4–6). In cases where neovascularization leads to PDR, characterized by the formation of abnormal blood vessel growth, photocoagulation stands as the current treatment option. Clinically, neovascularization is identifiable by fine loops or blood vessel networks on the retinal surface extending into the vitreous cavity, often appearing immature and regressing, resulting in ischemia and further neovascularization. The pathophysiology of DR is intricate, involving various cell types and molecules, as evidenced by numerous studies in both human and animal models (**Figure 1**). This review delves into distinct pathophysiological aspects of DR, along with identifiable cell types and molecules that could serve as targets to alleviate the chronic changes occurring in the retina due to diabetes mellitus. Identifying additional targets is significant in expanding the range of treatment options available for effectively managing DR.

## Blood retinal barrier breakdown in diabetic retinopathy

The blood-retinal barrier (BRB) is the primary defense for the retinal cells against the external factors damaging them. The inner BRB forms tight junctions between retinal capillary endothelial cells, while the outer BRB forms tight junctions between retinal pigment epithelial cells (7). The inner BRB layer contains endothelial cells merged in tight junctions and regulated by pericytes, which regulate transport through retinal capillaries within the inner retina (8, 9). Since BRB is a part of the neurovascular unit, a complex multi-cellular structure consisting of vascular cells (endothelial cells, pericytes), neurons (ganglion, bipolar, horizontal, and amacrine cells), glia (astrocytes, Müller cells, and microglial cells) and smooth muscle cells, maintaining its integrity during highly altered metabolic conditions of diabetes is critical for preventing BRB breakdown (10). Indeed, the loss of BRB integrity is the primary feature of DME. To begin with, in the presence of high levels of mediators that promote inflammation and activated microglia, VEGF is known to be released by macroglia, thus compromising the integrity of the barrier (11). Subsequently, endothelial cells begin to express cell adhesion molecules (CAMS) such as ICAM-1, VCAM-1, E-selectin, and P-selectin (12), a few of which have been linked to the cause of NPDR (13). On the luminal side of the endothelial cell anchors provided by CAMs, circulating leukocytes bind to the vessel wall through CD11a, CD11b, and CD18 (14) and release pro-inflammatory cytokines. This results in a culmination of cytotoxic mediators adding to the local pro-inflammatory climate (15) and weakening barrier integrity by compromising endothelial tight junction and pericyte-endothelial health (16). Additional studies have shown that loss of integral membrane protein caveolin-1 causes BRB breakdown, venous enlargement, and mural cell alteration (17). A possible target to prevent BRB breakdown is inhibiting cell adhesion molecules or integrins. This is supported by the fact that BRB breakdown significantly decreases when CAMs and integrins are inhibited or genetically deleted (14, 18). Indeed, increased VEGF levels in diabetic mice were shown to be directly correlated with increased levels of ICAM-1 and a loss in BRB, while blockade of VEGF suppressed such BRB breakdown (19). Also, increased VEGF levels activate protein kinase C, which in turn may directly or indirectly increase occludin phosphorylation, leading to its internalization and causing the breakdown of BRB (20).

Blood retinal barrier integrity is safeguarded by suppressing cytokine and chemokine activity (11, 21). The significance of the immune response in BRB breakdown has also been successfully proven in studies that combat inflammatory mediators and the complement system (22, 23). Chronic hyperglycemia polarizes microglia into a proinflammatory subtype through extracellular-signal-regulated kinase 5 (ERK5) (24), the levels of which were shown to be increased in the retina of STZ-induced DR model (25). Such increased ERK5 is known to elicit the production of cytokines such as IL-6, IL-1β, and VEGF. In turn, these inflammatory markers are known to impair vascular permeability. Thus, blocking the inflammatory pathways downstream of ERK5 through a small molecule inhibitor XMD8–92 prevented retinal inflammation, oxidative stress, VEGF production, and retinal vascular permeability (25). Apart from microglia, other immune cells such as the circulating T helper-17 (Th 17) in the STZ model of DR, induce IL-17A production. Few of the Th17 cells in the circulation were also shown to adhere to the retinal vasculature, thus predicting to be participating in the breakdown of BRB. The IL-17A secreted into the retina binds to their receptors on Müller glial cells and photoreceptors, activating the NF-κβ pathway, Fas-associated death domain (FADD) retinal vascular endothelial cell death, as well as ERK-dependent oxidative stress resulting in retinal vascular impairment and BRB dysfunction (26, 27). The role of photoreceptor cells in maintaining inner BRB in the diabetic retina is another emerging field. Photoreceptors in STZ-induced diabetic mice produce soluble factors, including ICAM1, inducible nitric oxide synthase (iNOS), and cyclooxygenase 2 (COX2), influencing inflammation in the neighboring leukocytes and endothelial cells to produce TNFα. Additionally, photoreceptor cells also release several inflammatory mediators such as IL-1α, IL-1β, IL-6, IL-12, TNF-α, chemokine C-X-C motif ligand 1 [CXCL1], CXCL12a, monocyte chemoattractant protein 1 [MCP-1], I-309, and chemokine ligand 25 [CCL25] impairing BRB permeability through partly modulating tight junction protein claudin (28, 29).

The outer BRB, formed at the retinal pigment epithelial (RPE) cell layer, controls the passage of solutes and nutrients from the choroid to the sub-retinal space but also plays a vital role under physiological conditions regulating vitamin A and protecting against oxidative damage. Additionally, RPE releases various growth factors and cytokines, including pigment epithelium-derived factor (PEDF), vascular endothelial growth factor (VEGF), fibroblast growth factor (FGF), insulin-like growth factor-I (IGF-I), tumor necrosis factor α (TNF-α), transforming growth factor β (TGF-β), interleukins (ILs) essential for retinal cell survival and immune privilege (30). Indeed, changes in the expression of these factors lead to destructive inflammation, neovascularization, and immune reactions under pathological conditions (31). For example, the essential role of PEDF, an anti-angiogenic factor in maintaining the integrity of outer BRB without affecting the structure and functionality of normal blood vessels, is known (32). Expectedly, PEDF therapy in diabetic mice reduces microgliosis, boosts tight junction expression, decreases pro-inflammatory mediator production, lowers vascular permeability, and is neuroprotective (8, 33). In addition to growth factors and cytokine release, RPE plays a vital role in solute transport through its tight junctions, occludin-1, claudins, and ZO-1, which are similar to those in other tissues (34). The cytokines released, in particular retinal IL-6 in DR, influence retinal vascular permeability (35) through microglial recruitment to the RPE layers disassembling tight junction complexes, including ZO-1 and occludin proteins (11). Additionally, IL-6-mediated microglial expression of TNF-α is shown to activate NF-κβ and thus reduce levels of ZO-1 in RPE cells. Indeed, STAT 3 inhibition reverses the disintegration of ZO-1, suggesting the essential role of IL-6 -STAT 3 pathways in microglial and RPE cells in regulating outer BRB (11). Lastly, the tight junctions, combined with the actin cytoskeleton, provide polarity to the RPE cells to regulate signal transduction and help localize certain proteins to maintain the outer BRB (36). Several studies using RPE cell lines have reported the benefits of maintaining the tight junctions as relevant to DR affecting tight junction permeability (e.g., C-reactive protein and tissue factor) and promoting tight junction formation (e.g., somatostatin, nicotinamide, lysophosphatidic acid, and HIWI2-mediated activation of Akt), highlighting their significance for maintaining epithelial phenotype (37). However, the relevance of these studies in animal models remains to be determined. Taken together with outer and inner barrier function, the integrity of the neurovascular unit of the retina is of primary importance to prevent BRB breakdown in preclinical DR and individuals with prolonged diabetes. Future studies exploring targets that could protect BRB will continue to be of interest to the field.

## Neovascularization in diabetic retinopathy

Neovascularization refers to the formation of new and abnormal blood vessel growth. It is one of the most common critical pathologies of DR. Clinically, neovascularization could be characterized by fine loops or blood vessel networks on the retinal surface extended into the vitreous cavity (38). Among the various growth factors identified, vascular endothelial growth factor (VEGF) has been well-established to play a central role in the neovascularization of DR (39).

VEGF is a homodimer glycoprotein that binds to heparin and has a molecular weight of 46 kDa. It is synthesized in human cells through alternative splicing. The transcription factor HIF-1 (hypoxia-inducible transcription factor 1) binds to the hypoxia-responsive enhancer elements (HREs) at the VEGF gene, upregulating transcription, which is physiologically regulated by oxygen tension (40, 41). Apart from enhancing the transcription of VEGF, HIF-1 also helps its stability by preventing VEGF mRNA degradation (42, 43). VEGF is biologically active through tyrosine kinase receptors (RTKs). VEGF family contains VEGF A, B, C, D and PlGF (Placental growth factor). VEGF receptors include VEGFR1, VEGFR2 and VEGFR3. VEGF-A can bind and activate VEGFR1 and VEGFR2, but PIGF and VEGF-B bind only to VEGFR1. VEGFA has 5 isoforms based on splice variants, and VEGF 165 is predominantly expressed in the retina (44). Additionally, it was shown that the transmembrane protein Neuropilin-1 functions as a coreceptor for VEGF-A (45, 46). Increased ischemia or hypoxia enhances VEGF production through hypoxia-inducible factor 1(HIF-1). Apart from endothelial cells, retinal Müller cells also produce significant amounts of VEGF in diabetic mice. This increase is associated with a three-fold increase in leukostasis and two-fold higher levels of ICAM-1 and proinflammatory marker TNF-α, drastic reduction in occludin and ZO-1 levels, ~60% increase in retinal vascular leakage in animal models. Conversely, inhibition of Müller cell-derived VEGF significantly reduces these effects, suggesting that VEGF production from Müller cells could be targeted to control DR (23). The irregular production and secretion of VEGF induce vascular endothelial cell proliferation and migration, increasing vascular permeability (39). Activating endothelial nitric oxide synthase (eNOS) and generating nitric oxide are further components of VEGF-A/VEGFR2’s control of vascular permeability and plasma extravasation (47). Multiple VEGF functions, such as survival, proliferation, migration, vascular permeability, and gene expression, have been mediated by activation of the phospholipase C, protein kinase C, Ca^2+^, extracellular signal-regulated protein kinase, mTOR, protein kinase B, Src family kinase, focal adhesion kinase, and calcineurin pathways (48).

Apart from VEGF, other key molecules involved in neovascularization in DR include platelet-derived growth factor (PDGF), placental growth factor (PlGF) and others. Platelet-derived growth factors are secreted by platelets, endothelial cells, activated macrophages and smooth muscle cells and are vital players of DR (49–51); PDGF has 4 polypeptide chains(PDGF-A, PDGF-B, PDGF-C, and PDGF-D) and becomes active by homodimer isoform formation such as PDGF-AA, PDGF-BB, PDGF-CC and PDGF-DD or heterodimeric form PDGF-AB. PDGF ligands will bind to transmembrane tyrosine kinase PDGF receptors, which contain homodimeric and heterodimeric isoforms such as PDGFR-αα, PDGFR-ββ and PDGFR-αβ (50). Retina-specific expression of PDGF-B could lead to neovascularization and retinal detachment (52, 53). In diabetic animal models, PDGF-AA and PDGF-BB levels were increased during the development of DR (54). Inhibiting PDGF-BB levels could prevent neovascularization (55). PDGF-CC could rescue neurons from apoptosis in the retina by regulating GSK3beta phosphorylation (56).

PIGF belongs to the VEGF family of growth factors. PlGF has 4 isoforms, and all its isoforms bind to VEGFR1 with different affinities than VEGF but do not bind to VEGFR2. Reports suggest that PIGF-1 is highly produced by RPE cells during pathogenesis, while low levels were detected under normal conditions (57–59), while PlGF-2 is produced by retinal endothelial cells and pericytes (60, 61). The 3D structure of PGF-1 shares 42% similarity in amino acid sequence with VEGF but has a strikingly similar 3D structure (62). Elevated levels of PlGF were observed in the aqueous humor and vitreous samples of DR patients and the levels were associated with retinal ischemia and VEGF-A levels (63, 64). While inhibition of VEGF increases PIGF levels, it also acts as a redundant inducer of neovascularization (65). Expression of PlGF is associated with several early and later features of DR in animal models (66). For example, inhibition of PlGF reduces neovascularization, retinal leakage, and associated inflammation and gliosis while preserving normal vascular development and neuronal architecture (65, 67).

Other than growth factors, Profilin1 (Pfn1), an actin-binding protein, was discovered through bioinformatic analysis to transcriptionally upregulated in vascular cells of patients with PDR and further confirmed in a mouse model that mimics PDR (68). Mechanistically, in the context of vascular development in the retina, the deletion of the Pfn1 gene in vascular endothelial cells postnatally impeded the formation of actin-based filopodial structures, tip cell invasion, vascular sprouting, and overall neovascularization, suggesting a crucial role for Pfn1 in promoting actin polymerization and angiogenesis (68). Inhibiting this Pfn1-actin interaction by a novel compound, C74, indeed proved to be a potential therapeutic target for conditions involving abnormal retinal angiogenesis as in PDR (69). Furthermore, the transcription factor FOXC1 has been found to be essential for normal revascularization processes, crucial for pericyte function, and vital for forming the blood-retinal barrier (BRB). Therefore, FOXC1 is now recognized as a therapeutic target for retinal vascular diseases such as DR. Specifically, the loss of FOXC1 in endothelial cells hindered retinal vascular development by reducing mTOR activity. However, treatment with the mTOR agonist MHY-1485 restored disrupted retinal angiogenesis (70).

## Microaneurysms in diabetic retinopathy

Microaneurysms represent a slight expansion of capillary walls, resulting from the excessive growth of endothelial cells (ECs) and depletion of pericytes due to prolonged unregulated high blood sugar levels, weakening blood vessel walls (71, 72). While the mean diameter of the normal capillaries is ~10µm (72), the mean diameter of microaneurysms in DR ranges from 43–266µm and span over more than one retinal layer (71–74). While intravitreal injection of VEGF into the eyes demonstrated microaneurysms, single and repeated anti-VEGF treatments reduced the microaneurysm levels significantly (71, 75, 76). Further, nearly 80% of the microaneurysms were identified near the capillary dropouts, representing focal ischemic regions (77). These studies suggest a direct link between focal ischemia, VEGF levels, and microaneurysms. However, microaneurysms are associated with resistance from anti-VEGF therapy as they are densely present in refractory areas, and more importantly, retinal thickness reduction after anti-VEGF treatment is minimal in these areas (78). Additionally, the areas with a higher density of microaneurysms were closely associated with residual edema after anti-VEGF treatment compared to areas with lesser edema (79).

Pericytes, dome-shaped cells found on the outer side of the basement membrane alongside endothelial cells, play crucial roles in maintaining capillary structure and function (74). Pericytes provide mechanical stability to the capillary wall and their recruitment depends on PDGFβ (80). Interacting with endothelial cells through paracrine signals and making direct cell-cell contact, pericytes in retinal capillaries assist in preserving barrier function (81). Indeed, pericyte density is higher in retinal vessels than in other capillaries to assist in upholding the barrier integrity (82). Persistent hyperglycemia induces transcription of angiopoietin-2 (Ang-2) mediated by tyrosine kinase receptor Tie-2, which provides signals to increase the number of migrating pericytes from the capillaries (83). Adequate endothelial secretion of PDGFβ is essential for pericyte function, as a decrease to less than half the normal pericyte density can lead to microaneurysm formation (84). Microaneurysms with pericytes tend to be smaller, though some contain inflammatory cells. Factors such as capillary nonperfusion, pericyte number (**Figure 1**), and inflammatory cells were all significant contributors to the size of microaneurysms (63). In animal models, the duration of diabetes correlates with increased acellular capillaries and pericyte loss (85). In DR, hyperglycemia-induced pericyte loss is primarily due to the inhibition of PDGF-BB/PDGFR-β downstream signaling through the activation of the PKC δ-p38 MAPK-SHP-1 pathway (86).

Signals from pericytes may be essential for endothelial cell survival (87). This includes the production of Angiopoietin-1 (ANG1, a heparin binding protein) (88), and VEGF-A (89). In DR, disrupted crosstalk between pericytes and endothelial cells leads to endothelial dysfunction and apoptosis (15). Furthermore, in diabetes, there is an upregulation of cPWWP2A expression in pericytes, affecting pericyte and vascular integrity. This dysregulation is mediated by miR-579 and its target genes, such as angiopoietin 1/occludin/SIRT1 (90). Overall, impaired pericyte-endothelial cell communications could lead to enhanced vascular permeability and leakage, and complications such as microaneurysms, macular edema, and neovascularization. Indeed, preventing early pericyte loss could be a possible approach to prevent complications associated with microaneurysms (90). Additionally, preserving pericyte-endothelial interaction could potentially mitigate neovascularization-induced retinal dysfunction (55, 91) by inhibiting PDGF, which is vital for pericyte function and neovascularization.

## Cell death in diabetic retinopathy

Neurodegeneration in the retina precedes vascular abnormalities, possibly leading to visual dysfunction in DR (92). In longitudinal examinations of type 1 and type 2 diabetes patients with no DR or mild NPDR, a progressive inner retinal layer thinning was found (93). The evolution of retinal thinning over time and the increase in the severity of the DR stage were also reported (94). Compared to individuals with normal glucose metabolism, reduced pericentral macula thickness was observed as early as in prediabetes conditions. A statistically significant linear trend was found to link macular thinning to the severity of glucose metabolism status (95). A further study showing thinner inner retinal layers and photoreceptor layers in patients with metabolic syndrome reinforced the notion that retinal neurodegenerative processes begin before the onset of DR (96). Retinal layer thinning may imply that variables other than hyperglycemia-induced thinning, such as insulin resistance and inflammation produced from adipose tissue, could impact neurodegeneration. In type 2 diabetic patients with early-stage DR, diabetes duration was inversely correlated with RNFL thickness, BMI, lipids, HDL, HbA1c, and albumin-creatinine ratio (97). Initial thickening of the macular ganglion cell inner plexiform layer (mGCIPL) and the rate at which the layer thinning was shown to be independent risk factors for developing DR (93). Additionally, a clear positive connection between loss of mGCIPL and decline in vascular density from baseline to 24 months was found in an investigation of eyes with no DR or mild NPDR. Thinner baseline mGCIPL and more considerable mGCIPL thickness reduction were substantially linked with a change in vessel density, according to multivariable regression analysis (93). These findings suggest that retinal microvascular integrity is related to retinal neurodegenerative characteristics.

**Figure 2:**
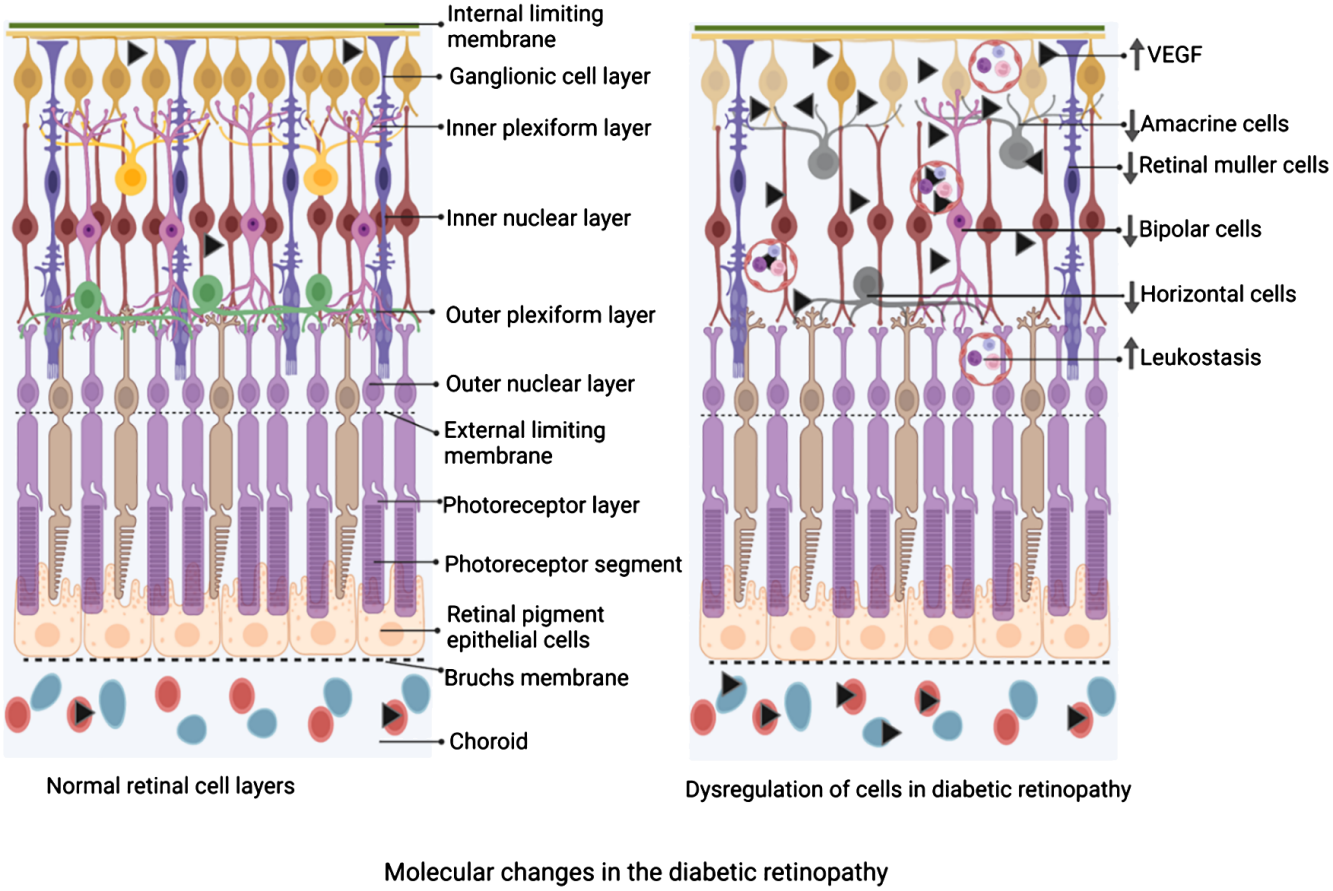
Schematic diagram depicting the changes in the cellular composition of the retina of diabetic retinopathy compared to the normal retina. On the left is a schematic of the layers of the retina under physiological state demonstrating an intact and significant number of Müller cells, horizontal cells, amacrine cells, bipolar cells, etc, while adherent leucocytes are not seen. On the right is a schematic of the layers of the retina under diabetic retinopathy. Note, a reduced number of Müller cells, horizontal cells, amacrine cells, and bipolar cells, while a significant number of adherent leukocytes are depicted. The figure was created using BioRender.

Neurodegeneration is a prominent aspect of DR (98–100) and may precede the clinical and morphometric vascular changes of diabetes (99). In the early stages of DR progression, apoptosis of retinal neurons reduces the thickness (**Figure 2**) of the inner retinal layers and the retinal nerve fiber layer (RNFL), as observed in optical coherence tomography (OCT) (101). Various types of retinal cells undergo death in DR (100). Different types of retinal cells undergo distinct forms of cell demise. Endothelial cells primarily experience apoptosis, while pericytes undergo apoptosis and necrosis, whereas Müller cells are expected to succumb to pyroptosis (100, 102–104). Additional mechanisms of endothelial cell death include hyperglycemia-induced reactive oxygen species (104) and excessive amounts of adherent leukocytes in DR (103). Retinal pericyte apoptosis has been linked to hyperglycemia-induced activation of NF-κB (105) and increased Bax levels, as observed in the human diabetic retina (106). Sorbitol is known to accumulate in the diabetic retina, and severe hyperglycemia-induced increased activity of the polyol pathway promotes retinal neuronal apoptosis and altered expression of GFAP (107).

In animal models, chronic diabetes reduces the thickness of inner plexiform and inner nuclear layers with significant ganglion cell apoptosis even in the early phases of diabetes. This early cell death is also evident in human retinas (99). Also, in the late chronic phase of DR, a significant amount of cell death was observed in untreated DR rats and DR rats treated with anti-VEGF drugs (108). In areas with more significant aggregation of activated microglia, spectral-domain optical coherence tomography found neuronal thinning in the retinas of diabetic rodents, consistent with this manifestation (109, 110). Microglia could destroy neurons and prune synapses, and their activation occurs before neuronal thinning, while they could also phagocytose damaged but functional neurons (111), which may help explain the faster loss of neurons in the early stage of the disease. Caspase activation in the human diabetic retina is well-known (112), and inhibition of caspase activation prevents capillary degeneration in DR (113). Also, inner retinal astrocyte dysfunction was demonstrated during early diabetes with neuronal functional loss (114). Also, prolonged increases in the levels of VEGF induce apoptosis. At the same time, anti-VEGF treatment is concomitant with reduced levels of VEGF and apoptosis (115). In DR, caspase 3 activated the progressive death of retinal ganglionic cells and other retina cell types mostly due to uncontrolled hyperglycemia-induced neurovascular complications (99, 100, 116–119). Retinal cell death in DR is also associated with significantly reducing GABA inhibitory neurotransmitter signaling (120, 121). Treatment with GABA analog pregabalin significantly suppressed retinal IL-1β, TNF-α, CD11b, caspase 3, oxidative stress, and glutamate levels, suggesting GABA requirement for retaining normal retinal function in DR (121). Direct controlled nano delivery of GABA to the retina for protecting RGCs in DR needs further evaluation as such deliveries significantly improved GABA deficiency symptoms in the brain (122).

Neurotrophic factors such as nerve growth factor (NGF), Brain-Derived Neurotrophic Factor (BDNF), Ciliary Neurotrophic Factor (CNTF), and Glial Cell-Derived Neurotrophic Factor (GDNF) in the retina are essential for the development and survival of retinal cells (123). NGF levels were significantly elevated in the serum and tear fluid of PDR patients compared to non-diabetic controls and NPDR patients (124). In DR, an increase in proNGF and a decrease in NGF were detected in the retina, correlating with a significant rise in retinal neuronal death (125). In the retinas of diabetic humans and rats, substantial levels of lipid peroxidation, nitrotyrosine, and the pro-apoptotic p75(NTR) receptor were observed. Hyperglycemia-induced peroxynitrite accumulation in the diabetic retina impairs NGF neuronal survival by nitrating the TrkA receptor and increasing the expression of p75(NTR) (126, 127). Peroxynitrite accumulation also hampers the production and activity of matrix metalloproteinase-7 (MMP-7), which extracellularly cleaves proNGF, activates RhoA/p38MAPK, and contributes to neurovascular death in the retina of DR patients and rat models (125). In a mouse model of PDR, early treatment with antagonists of p75(NTR) or proNGF or NGF inhibitors suppressed retinal neuronal cell death and other pathologies, indicating potential additional therapeutic targets for DR (128, 129). Indeed, it was shown that twice-daily treatment with NGF eyedrops in DR rats significantly prevented retinal ganglion cell death (130). Remarkably, a high molecular weight protein like NGF can be transported from the anterior to the posterior segment to reach the retina and optic nerve, providing protection Serum and aqueous humor levels of BDNF were significantly lower in diabetes mellitus patients before DR development (131). Additionally, BDNF levels were significantly reduced in the retina of DR rats (132, 133). BDNF protects against neurodegeneration in DR, and the neuroprotection and Müller cell viability it offers are mediated by VEGF (134). Syn3, a BDNF signaling enhancer, significantly reduced retinal ganglion cell loss in DR 10 weeks after intravitreal injection, suggesting a new approach for preventing DR progression (135). However, conflicting reports exist, with studies showing higher levels of NGF, BDNF, GDNF, NT3, NT4, and CNTF in the vitreous of DR patients (136, 137) and elevated levels of NT3 and NT4 in the retina of DR rats (137). In summary, although several neurotrophins in the retina were shown to be essential for the development and survival of retinal cells, the specific roles of individual neurotrophins on retinal cell survival require further investigation.

## Leukostasis in diabetic retinopathy

Leukostasis has been documented in both animal models and human subjects with DR (13). Leukostasis involves the closure of the retinal microvasculature and endothelial cell death temporally and spatially associated with adherent leukocytes in DR (103). Leukocyte endothelial interactions, which show the escalating inflammatory response, occur intravascularly during the early phases of DR. Leukostasis is brought about by activating myeloid cells like neutrophils, monocytes, and granulocytes. Leukostasis is identified by immune cells stuck in the narrow retinal blood vessels, leading to blockage and lack of blood flow. In the microcirculation, the presence of rolling and adherent neutrophils, their velocity of movement, and the areas affected by leukostasis worsen with the severity of hyperglycemia (138). Also, increased leukostasis was demonstrated in different animal models of DR with short-term and long-term studies (139). In advanced stages of DR, there is a rise in the systemic neutrophil count, and these cells exhibit heightened expression of myeloperoxidase and produce greater amounts of hydrogen peroxide compared to cells sampled from individuals without diabetes (140).

Leukocyte-endothelium adhesion is characterized by increased production of endothelial cell adhesion molecules and integrins. Leukocytes engage in a multi-step process on the surface of endothelial cells, where they interact with these molecules to stick to the endothelial wall. For example, intercellular adhesion molecule-1 (ICAM-1), vascular cell adhesion molecule-1 (VCAM-1), and selectins, as well as leukocyte b2-integrins (CD11a, CD11b, and CD18) (E-selectin) were shown to be elevated in DR. In human subjects with DR soluble E-selectin and sVCAM-1 as well as CCL17, CCL19, and TGF-β were shown to be elevated (141). Indeed, the DR severity was correlated to the plasma levels of VCAM-1 and E-selectin (142). In DR rats, a significant increase in the capillary occlusions was noted due to granulocytes and monocytes associated damage to endothelial cells, thus accumulating extravascular leukocytes, neovascularization, and tissue damage (143). Not surprisingly, diabetic rats demonstrated significantly increased leukostasis in the chronic phase of DR (108).

A variety of molecules and processes have been implicated in higher levels of leukostasis in DR. VEGF has been shown to promote ICAM-1 expression in endothelial cells, thus triggering leukocyte activation and cytokine release, which in turn creates amplifying inflammatory response and increasing VEGF expression. In the early stages of DR progression, the specific endogenous VEGF inhibition has been shown to reduce retinal leukostasis and BRB breakdown, attesting to VEGF’s prominent role in leukostasis (19). Additional studies using sulfonated oligosaccharides to inhibit VEGF in the retina in diabetic rats resulted in the inhibition of leukostasis and improved ERG (144). Leukocyte-induced microvascular damage by physically blocking capillaries results in temporary ischemia, resulting in increased VEGF or the release of cytokines and superoxide through the respiratory burst (145). The renin-angiotensin system, oxidative stress, and various other abnormalities associated with diabetes are recognized factors that elevate leukostasis within the retinal blood vessels of diabetic rats, mice, and monkeys. Indeed, enhanced intravascular polymorphonuclear leukocyte counts have been observed around areas of capillary nonperfusion in the retinas of diabetic monkeys (146, 147). In animal models, the deletion of ICAM-1 and CD-18, essential for white blood cell adherence to the endothelium, greatly slowed down diabetes-induced capillary degeneration (103). Leukocytes from diabetic rats, but not control rats, caused *in vitro* endothelial cell death, attesting to the ability of activated leukocytes to damage the endothelial wall (147). The increased Fas (CD95)/Fas-ligand pathway is associated with elevated leukostasis to endothelial dysfunction and BRB impairment. Finally, inducible nitric oxide synthase (iNOS) isoform is demonstrated as a key mediator of leukostasis and BRB breakdown in DR (148).

## Retinal inflammation in diabetic retinopathy

Inflammation in the retina is directly linked with the severity of DR (149, 150). However, to date, no single proinflammatory molecule has been exclusively associated with the progression of DR. Using both genetic and induced animal models of DR, it was demonstrated that low-grade subclinical inflammation promotes multiple vascular complications such as pericyte and endothelial cell loss, formation of acellular capillaries, and thickening of the basement membrane of retinal vessels in DR (18, 145). While hyperglycemia is directly linked with increased levels of pro-inflammatory cytokines (151), inflammation in DR is known to persist from the early stages of diabetes to the vision-threatening form of the disease (145).

Tumor necrosis factor (TNF-α), a widely recognized cytokine associated with inflammation, has been demonstrated to have negative implications in DR (152–156). TNF-α triggers alterations in endothelial cells, notably promoting the expression of intercellular adhesion molecule ICAM-1, which is crucial in recruiting leukocytes. Other than TNF-α, elevated levels of pro-inflammatory cytokines such as IL-1β, and IL-6 and chemokines like MCP-1, CCL2, and CCL5 were documented in mouse models (153). Not only ICAM-1 but also elevated levels of VCAM-1, draw monocytes and leukocytes and encourage an ongoing inflammatory response (157–159). Inflammatory cells invade, and damage tissues as chronic inflammation builds up, increasing retinal vascular permeability, vasodilation, and retinal thickness in DR subjects. Increased levels of TNF-α, IL - 1β, IL - 1α, rantes and MCP-1 were also demonstrated in the serum/aqueous humor of DR patients (160, 161). While the elevated levels of these cytokines might have many downstream effects, increased TNF-α and hyperglycemia are known to cause endoplasmic reticulum (ER) stress in retinal endothelial cells (162). Interestingly, the elevation of ER-specific protein, glucose-regulated protein (GRP78) at the plasma membrane in endothelial cells interacts with the VE-Cadherin, a junction protein in the endothelium that is required for cell-to-cell adhesion in the blood vessels glycosylating VE-Cadherin (GlcNAcylated VE-cadherin), thus increasing transmigration of leukocytes across endothelium and increased permeability creating a perpetual motion of inflammation and ER stress (163).

In both the serum and retinal Müller cells, IL-33 levels are elevated in diabetic conditions (164, 165), whereas IL-35 levels in the vitreous are decreased in diabetic retinopathy (DR) (166). In the peripheral blood mononuclear cells of patients with proliferative diabetic retinopathy (PDR), IL-35 lowers IL-17 levels and inhibits Th17 cell differentiation, offering protection against PDR (167). The proinflammatory cytokine IL-17 is elevated in the plasma and vitreous of diabetic patients, and the worsening of DR is linked to increased retinal IL-17A expression through Act1 signaling, which leads to Müller cell dysfunction (168, 169), and promotes retinal neovascularization (170). In PDR, vitreous IL-17A levels are associated with IL-10, IL-22, and TNFα levels in the aqueous humor and vitreous (171). Administering intravitreal or intraperitoneal injections of anti-IL-17A antibody or anti-IL-17RA antibody to type 1 and type 2 diabetic mice significantly reduces DR pathologies, including Müller cell dysfunction, leukostasis, leakage, downregulation of tight junction proteins, and ganglion cell apoptosis in the retina (27, 172).

Other than pro-inflammatory cytokines, various other molecules are well-known to be involved in DR. For example, in diabetes patients, elevated intracellular glucose levels were known to activate the polyol pathway, which metabolizes glucose (173), resulting in elevated deposition of AGEs. This increased AGEs activates protein kinase C (PKC), AGE receptor upregulation, and overactivity of the hexosamine pathway (174). This, in turn, increases the reactive oxygen species (ROS) within cells, resulting in irreversible cellular damage and chronic inflammatory stress (175). Chemokines that control the leukocyte recruitment and activity play an essential role in the development of DR. To this end, monocyte chemotactic protein-1 (MCP-1) and macrophage inflammatory protein-1 alpha (MIP-1α) have been shown to be higher in diabetic individuals (176, 177).

The retinal glial cells, which encompass supportive structural elements such as astrocytes, Müller cells, and microglial cells, play a vital role in maintaining cellular equilibrium. The development and advancement of retinal inflammation in DR are associated with dysfunction within the retinal neuroglia (178). Research from both laboratory experiments conducted *in vitro* and studies utilizing animal models and human post-mortem samples have indicated that the activation of retinal microglia may play a critical role in modulating cytokine expression. This regulation of cytokine expression by activated retinal microglia could have substantial implications in regulating retinal inflammation associated with diabetes (179). In the presence of hyperglycemia, glial cells exhibit dysfunction, leading to an imbalance in oxidative stress and levels of pro-inflammatory cytokines such as TNF-α, growth factors, IL-1, and IL-6. Retinal evaluation in the early experimental diabetes models also revealed selective and progressive accumulation of FDP-lysine. This, in turn, leads to Müller glial cell dysfunction and upregulation of VEGF, IL-6, and TNF-α., providing evidence of the contribution of advanced lipoxidation end-product formation for the retinal inflammation and pathogenesis of DR (180). Moreover, the pro-inflammatory cytokines released from glial cells contribute to migrating monocytes and T lymphocytes. Persistent inflammation further triggers fibrotic processes, leading to the formation of scar tissue, which can ultimately result in retinal detachment (119). While VEGF is a known inducer of inflammation, various other modulators are also known to enhance retinal inflammation independent of VEGF (181). Equally, inflammation is also known to mediate angiogenesis in DR (182) while the interaction of CD40 Ligand with CD40 is an intermediate step between Inflammation and angiogenesis in DR (183).

## Advancing therapies for treating diabetic retinopathy

Over the years, several therapeutic approaches have been developed to manage DR, ranging from conventional treatments to cutting-edge advancements in the field of ophthalmology (**Table 1**). Some of the main modes of treatment available for DR are photocoagulation, vitrectomy, steroid, and anti-VEGF therapies. Two main types of lasers are used in photocoagulation: pan-retinal photocoagulation (PRP) targets leaking blood vessels, while focal laser therapy targets specific areas of abnormal growth. These procedures can slow DR progression but may not always restore vision. In severe cases such as in PDR with bleeding or scar tissue in the vitreous, vitrectomy may be necessary to remove these obstructions and improve vision. Corticosteroids can reduce inflammation in the retina, while effective in some cases, their use is limited due to potential side effects like cataracts and glaucoma.

**Table 1:** Salient exploratory therapeutic targets for DR.

Drug/protein/Biologics	Target	Clinical/preclinical studies and indications	References
Bevacizumab (Avastin)	VEGF-A	Off-label drug for DR, FDA-approved for neovascular (wet) age-related macular degeneration (AMD) and macular edema following retinal vein occlusion	(184)
Ranibizumab (Lucentis)	VEGF A	FDA-approved for the treatment of DR in patients with DME and for other eye conditions such as neovascular AMD and macular edema following retinal vein occlusion.	(185)
Aflibercept (Eylea)	VEGF B, PIGF 1, PIGF-2	FDA-approved for DR in patients with DME and other eye conditions such as neovascular AMD and macular edema following retinal vein occlusion.	(186)
Faricimab (VABYSMO)	VEGF-A and angiopoietin-2 (Ang-2).	FDA-approved for neovascular (wet) age-related macular degeneration (nAMD) and DME in clinical trials for DR	(187)
Brolucizumab (Beovu)	VEGF	FDA-approved for the treatment of neovascular (wet) age-related macular degeneration (AMD) and in clinical trials for DME and DR.	(188)
Infliximab	TNF-α	Clinical studies: Decreased macular thickness and improved visual acuity in a diabetic model.	(189)
SAR 1118	antagonist of LFA-1	Preclinical studies: Reduced leukostasis and retinal vascular leakage. Potential therapeutic for DR or other retinal vascular disorders.	(190)
Losartan, an AT1R blocker, or Enalapril, an angiotensin-converting enzyme inhibitor	RAS	Clinical studies: Reduced progression of retinopathy by 70%, while treatment with Enalapril reduced it by 65% in a clinical trial involving type 1 diabetes patients with normotensive and normoalbuminuria. RAS blockade is potentially therapeutic in preventing or delaying the development of DR.	(191)
Pigment Epithelium-Derived Factor (PEDF)	PEDF	Preclinical studies: PEDF overexpression prevented neovascularization in a murine adult model of retinopathy, indicating a protective effect against abnormal blood vessel growth in the retina, a hallmark of DR and age-related macular degeneration.	(192)
miR-182–5p	angiogenin and BDNF	Preclinical studies: miR-182–5p exerts an inhibitory effect on retinal neovascularization, indicating its regulatory role in the formation of abnormal blood vessels in the retina, which is a characteristic feature of diabetic retinopathy and retinopathy of prematurity	(193)
Angiopoietin-like 4	ANGPTL4	Preclinical studies: Gene therapy mediated modulation of ANGPTL4 expression, a potential therapeutic approach for stabilizing blood vessels and reducing vascular leakage in diabetic retinopathy.	(194)
Syn3	BDNF enhancer	Preclinical studies: Syn3 demonstrated significant protection against RGC loss in DR	(135)
Anti-IL17A	IL 17-A	Preclinical studies: anti 1L-17A injection halted diabetes-mediated retinal inflammation, vascular impairment, and the onset of diabetic retinopathy (NPDR)	(27)
UPARANT	Urokinase receptor-derived peptide inhibitor	Preclinical studies: Protected the BRB integrity and prevented neovascularization in DR.	(195)
XMD8–92 {2-[[2-Ethoxy-4-(4-hydroxy-1-piperidinyl)-5,11-dihydro-5,11-dimethyl-6H-pyrimido[4,5-b] [1,4]benzodiazepin-6-one}	ERK 5	Preclinical studies: XMD8–92 reduced diabetes mediated retinal inflammation, oxidative stress, vegf production, capillary degeneration and vascular leakage	(25)

Anti-VEGF therapy through intravitreal injections is preferred for treating DME associated with vision loss (196–198). Several anti-VEGF drugs, including Bevacizumab, Ranibizumab, Aflibercept, Faricimab, and Brolucizumab have been used for the treatment of DR. These agents improve visual acuity and reduce retinal thickness due to edema. They continue to be frontline therapies, building on results from landmark trials. Bevacizumab, initially developed for cancer therapy (199), has shown efficacy in treating DR and DME (184, 200). Ranibizumab was the first FDA-approved anti-VEGF protein for treating DME in 2012. It is a 48 kDa monovalent monoclonal antibody designed for ocular use and binding (201). The small size and lack of the Fc domain of this drug increase the penetration of the drug within the choroid and retina (202, 203). Phase 3 clinical trials showed that patients with monthly ranibizumab gained ≥15 letters at 2 years, with higher structural improvement in optical coherence tomography and resolution of leakage. They were also less likely to develop PDR (6, 185). Aflibercept, also known as VEGF trap, is a 115-kda dimeric glycoprotein and acts as a decoy receptor for VEGF isoforms -A& B and PLGF. Its improved binding properties help to reduce treatment burden and follow-up visits (186). Faricimab is a humanized antibody that targets both VEGF-A and angiopoietin-2 (Ang-2). This multitarget profile presents intriguing new options for treating exudative retinal disorders. In light of this, Faricimab’s Promising data on the use of Faricimab in DME was provided by the phase 2 BOULEVARD study, which demonstrated its superiority over ranibizumab in terms of visual gain (187). Further, in a phase 3, prospective, randomized, double-masked, multicenter study, Brolucizumab demonstrated greater fluid resolution compared with aflibercept (188). Despite the advances with anti-VEGF therapies, ~40% of cases are refractory with poor response to this treatment (4–6, 196), indicating the requirement of additional dossing or developing new targeting for treating DR. To this end, repeated intravitreal injection of these drugs demonstrated significant improvement in the visual acuity and retinal thickness across studies or delivery of anti-VEGF drugs in nanoformulations reduced the frequency of intravitreal injections in animal studies (108). Apart from VEGF, other growth factors, including placental growth factor (58–60), platelet-derived growth factor (55, 91), and nerve growth factor (204), are known to play important roles in neovascularization and neuronal loss. Individual or combined inhibitions of these growth factors may provide optimal outcomes.

As consistent cell death occurs in different retina layers, neurodegeneration could be a major factor in vision loss. Neuroprotective strategies may be required to maintain the sustained cell density of the retina in DR. Protecting retinal neuronal cells from damage caused by diabetes is another promising area of research. These drugs may help preserve vision even if blood vessel abnormalities persist. For example, diabetic mice demonstrating elevated NLRP3 inflammasome activation and increased production of IL-1β by Müller glia could be abrogated with Müller glia-specific Regulated in Development and DNA damage 1 (REDD1) deletion, thus improving vision (205). In another example, changes in the microglial immune ligand-receptor CD200-CD200R complex were shown to be associated with neuroinflammation in DR, and CD200Fc, a CD200R agonist, effectively mitigates microglial activity, providing a novel immunotherapeutic target for treating DR (206). Finally, glucagon-like peptide-1 (GLP-1) has been shown to reduce the intracellular overload of Ca^2+^ influx through voltage-gated Ca^2+^ channels, thus protecting RGCs against excitotoxic Ca^2+^ overload in an STZ-induced diabetic animal model (207).

As persistent oxidative stress and inflammation initiate and promote multiple molecular changes, sustained inhibition of molecular pathways to block oxidative stress and inflammation in the retinal tissue could provide an essential avenue in developing therapies. For example, an increase in Takeda G protein-coupled receptor 5 (TGR5) receptor signaling in diabetes is associated with an increase in inflammation and ER stress in the retina and is decreased upon treatment with tauroursodeoxycholic acid (208, 209). ER, stress is also known to augment 12/15-LO-induced retinal inflammation in DR via activation of NADPH oxidase and VEGFR2, and thus, perturbation of the 12/15-LO pathway could help develop DR therapies (210). In a clinical investigation including four patients who did not improve after laser photocoagulation treatment, Infliximab, a TNF-α neutralizing antibody, was shown to decrease macular thickness to enhance visual acuity (189). In a diabetic rat model, topical administration of SAR 1118, a minor antagonist of LFA-1(leukocyte function associated antigen-1) expressed in leukocytes, decreased leukostasis and retinal vascular leakage in a dose-dependent manner (190). Apart from these distinct inflammatory molecules, the AGEs/RAGE pathway is also a potential target for DR due to its prominent role in inducing retinal inflammation. In animal models of DR, soluble RAGE blocking the RAGE activation improved neuronal dysfunction, thus reducing the acellular capillaries and pericyte ghosts (211). Blockade of RAS with Losartan, an AT1R blocker, or Enalapril, an angiotensin-converting enzyme inhibitor, significantly reduced the progression of retinopathy by 70% and 65%, respectively, in a clinical trial involving type 1 diabetes patients with normotensive and normoalbuminuria (191). Since NADPH oxidase activity is dysregulated in DR and contributes to oxidative stress and inflammatory cascades, blocking NADPH oxidase was shown to reduce oxidative stress, NF-κB activation, reactive NOS production, and inflammatory responses in retinal cells treated with high glucose (212, 213). Also, intravitreal injection of tissue inhibitor of matrix metalloproteinase-3 (TIMP-3) prevented BRB breakdown in diabetes and downregulated NF-κB, ICAM-1 and VEGF (214). Urokinase plasminogen activator (uPA)/uPA receptor (uPAR) system is another pathway that was shown to disrupt BRB in DR (215, 216). uPA inhibition with a peptide inhibitor UPARANT significantly protected the BRB integrity and prevented neovascularization in DR rats (195). Additionally, UPARANT also modulates transcription factors responsible for inflammation (217).

Lastly, anti-inflammatory drugs that have demonstrated a significant reduction in inflammation associated with other complications of diabetes mellitus could be evaluated for DR (218, 219). Similarly, regular use of plant-based phytochemicals and dietary supplements with antioxidant and anti-inflammatory properties modulates persistent retinal oxidative stress and chronic inflammation in the retina and prevents DR progression (220–224). Recently, Esculeoside A (ESA), a tomato-derived glycoside, has been shown to alleviate retinopathy in an *in-vivo* rat model of T1DM. The protective mechanism is found to be mediated by the Nrf2/antioxidant axis (225). Overall, research on the drugs that could reduce oxidative stress and inflammation likely help maintain the cellular and molecular integrity of the neurovascular unit, and research along these lines is necessary to prevent complications and vision loss associated with DR.

The research landscape continues to evolve, with promising new avenues, such as gene and cell therapies, which may offer new avenues for managing diabetic eye disease. Gene therapy for diabetic retina employs gene-specific targeted therapy, which is split into two categories based on the pathophysiology of the disease (226): therapies that target pre-existing neovascularization such as the use of sFlt-1, a soluble splice variant of the VEGF receptor 1 (VEGFR-1 or Flt-1), that acts as a decoy VEGF receptor and vascular hyperpermeability (227), and therapies that try to prevent damage to retinal blood vessels such as pigment epithelium-derived factor (PEDF) (192), angiogenin (193), and glial fibrillary acidic protein (GFAP) (228) and those that protect neurons such as the AAV vectors encoding brain-derived neurotrophic factor (BDNF) (229) and erythropoietin (EPO) (230). Some research suggests that modulating ANGPTL4 expression through gene therapy could help stabilize blood vessels and reduce vascular leakage in DR (194). Clinical trials are needed to evaluate the feasibility and effectiveness of this approach. Exploratory cell therapies independent of gene therapies in DR involve the transplantation or manipulation of cells to address the underlying pathology of the disease. Some of these approaches include mesenchymal stem cells (MSCs) (231, 232) or induced pluripotent stem cells (iPSCs) (233, 234) for their regenerative properties to replace damaged cells, promote tissue repair, or modulate the inflammatory response associated with the disease. Cell therapy approaches involving the transplantation or stimulation of endothelial progenitor cells (EPCs) and/or endothelial colony-forming cells (ECFCs) aim to enhance vascular repair mechanisms and improve blood vessel function in the retina (235). Clinical trials are ongoing to assess the safety and efficacy of EPC-based therapies in DR patients (NCT02119689).

## Conclusions and future directions

In summary, the future of DR and DME management looks promising, with ongoing trials aiming to advance treatment options and improve visual outcomes. Despite the tremendous progress in understanding various cell and molecular targets in DR, several challenges need to be addressed soon in the near future. For example, DR involves multifaceted processes, including inflammation, angiogenesis, and neurodegeneration. Pinpointing specific targets within this intricate web of interactions can be challenging. Additionally, DR progresses over time, with varying molecular profiles at different stages. Identifying targets that remain relevant throughout disease progression is essential. Likely, single-target approaches may not suffice. Combining multiple therapies could yield better outcomes. We hope that as research progresses, personalized approaches and innovative treatments may transform the management of this sight-threatening condition.

## Author contributions

SR: Writing – original draft, Writing – review & editing. VD: Supervision, Writing – review & editing. ATMS: Writing – original draft, Writing – review & editing. SS: Supervision, Writing – original draft, Writing – review & editing. KMRB: Supervision, Writing – review & editing. RG: Conceptualization, Supervision, Writing – original draft, Writing – review & editing. DU: Conceptualization, Supervision, Writing – original draft, Writing – review & editing.
